# Serologic Responses to COVID-19 Vaccines in Hematological Patients Are Predominantly Impaired in Lymphoid but not in Myeloid Malignancies

**DOI:** 10.1097/HS9.0000000000000686

**Published:** 2022-02-15

**Authors:** Verena Petzer, Normann Steiner, Olga Angelova-Unterberger, Gabriele Hetzenauer, Kathrin Philipp-Abbrederis, Ella Willenbacher, Clemens Feistritzer, Wolfgang Willenbacher, Jakob Rudzki, Reinhard Stauder, Florian Kocher, Andreas Seeber, Andreas Pircher, Piotr Tymoszuk, Christian Irsara, Alexander Egger, Vilmos Fux, Markus Anliker, Eberhard Gunsilius, David Nachbaur, Stefan Schmidt, Dominik Wolf

**Affiliations:** 1 Department of Internal Medicine V (Hematology and Oncology), Comprehensive Cancer Center Innsbruck (CCCI), Medical University Innsbruck (MUI), Innsbruck, Austria; 2 Data Analytics As a Service Tirol, DAAS Tirol, Innsbruck, Austria; 3 Central Institute of Clinical Chemistry and Laboratory Medicine Medical University of Innsbruck, Austria

The global COVID-19 pandemic caused by SARS-CoV-2 is still ongoing and challenges societies and their health systems. While overall mortality rate is 1%–3% in the general population, death rates are up to 37% among hematological patients.^1,2^ These patients are prone to severe COVID-19 associated complications, for example, higher hospitalization and invasive ventilation rates. Considering the constant appearance of new mutations with potentially higher infection and mortality rates, and the fact that vaccination represents the most effective preventive measure for severe COVID-19, identification and prioritization of vulnerable patients is of utmost importance.^3,4^ In most hematological patients, a compromised immune system due to the disease per se, the treatment, or a combination of both, might be responsible for an increased risk for severe or even life-threatening COVID-19. Thus, identification of patient subgroups with appropriate vaccination responses is of clinical relevance. Numerous studies have meanwhile shown that immunocompromised (including hematologic or oncologic) patients have a risk to completely fail or mount only a suboptimal humoral immune responses to SARS-CoV-2 vaccines.^5–8^

At the time when COVID-19 vaccines became available for hematological patients in Austria, studies had not been published. At our department all hematological patients, irrespective of the underlying disease, current therapy status or age were vaccinated. Pursuing this strategy, we conducted a retrospective analysis in adult patients with malignant hematological diseases who received two doses of either BNT162b2 (Biontech/Pfizer), mRNA-1273 (Moderna Biotech) or AZD1222 (AstraZeneca) between January 2021 and May 2021 (Suppl. Figure S1A and B and Table S1). The trial protocol was approved by the ethics committee at Innsbruck Medical University (approval: 1331/2021).

In total, we analyzed n = 123 patients, of these n = 43 patients suffered from myeloid, n = 63 from lymphoid malignancies, and n = 17 have undergone allogeneic peripheral blood stem cell transplantation (allo-SCT) (Suppl. Figure S1A). Antibodies against the nucleocapsid protein and the receptor-binding domain of the spike protein of SARS-CoV-2 were detected in serum using the Roche Elecsys Anti-SARS-CoV-2 assay on the Cobas e602 platform and the Abbott SARS-CoV-2 IgG II Quant assay on the ARCHITECT i platform, respectively (for details, see Suppl. Digital Methods). After the second vaccine dose (median: 35 d; interquartile range: 25–52 d) 102 patients (82.9%) mounted a humoral response to vaccination [defined by >7 binding antibody unit (BAU)/ml]. Patients with lymphoid malignancies have a significantly lower response rate as compared to myeloid malignancies or allo-SCT (71.4% lymphoid, 97.7% myeloid, and 88.2% allo-SCT, *P* = 0.0068; Figure 1A). Antibodies against the nucleocapsid protein were seen only in few patients (n = 4/2/2 in myeloid/lymphoid/allo-SCT), all of them mounting a vaccination response, excluding that differences between the groups are due to imbalances in previous SARS-CoV-2 infection.

**Figure 1.**
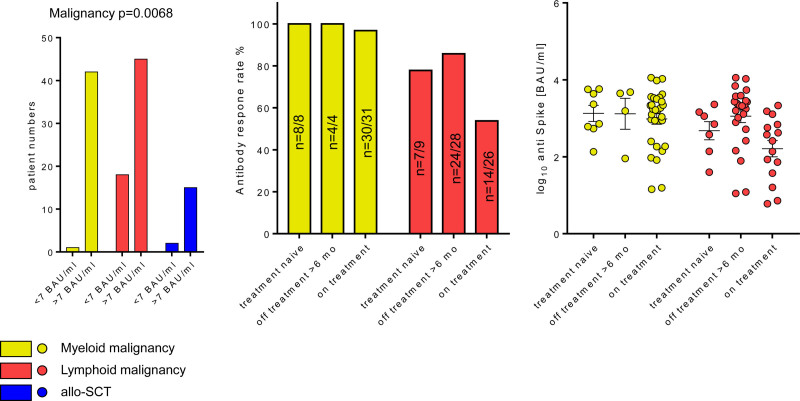
**Humoral vaccination responses according to disease and treatment.** Frequency of positive vaccination response (>7 BAU/mL) according to underlying malignancy (A) and treatment status (B). (C) Antispike antibody titer in the individuals with positive antibody response (C) was investigates in the study participants stratified by malignancy type and therapy status. Statistical significance was determined by χ2 (A) and one-way ANOVA and corrected for multiple testing with Benjamin Hochberg method (C). Significant test *P* values are presented in the plot captions.

As treatment status has been found to be a relevant predictor for serological response rate to SARS-CoV-2-vaccination, we further subdivided the disease cohorts into the following categories: “treatment naive,” “off treatment > 6 months,” and “on therapy” (ie, currently receiving or having received therapy within the last 6 mo). Therapy status neither affected antibody titer width nor the overall vaccination response rate (Suppl. Figure S2B). However, in the lymphoid malignancy subset, we could observe a tendency towards lower SARS-CoV-2 vaccination response in patients under treatment (53.8% vs 77.8% in naive and 14.3% in therapy-off, Figure 1B). In contrast, patients with myeloid malignancies have a favorable humoral response rate, despite ongoing therapy.

The negative correlation of lymphoid disease with positive response rate was corroborated by univariate logistic modeling (odds ratio [OR]: 0.0595; 95% confidence interval [CI] 0.0032–0.308 vs. myeloid; Figure 2A, Suppl. Table S3). In addition, lymphoid disease patients tended to correlate with lower antibody titers (β: −0.26, 95% CI −0.59 to 0.072 vs myeloid, Figure 2B) and this phenomenon was especially evident in patients undergoing treatment during vaccination (Figure 1C). This underscores that patients suffering from lymphoid malignancies have an impaired vaccination response. To which extent this translates into an increased risk of infection and/or severe COVID-19 remains unclear, as it is currently unknown which antibody titers confer sufficient protection. Antibody responses to SARS-CoV-2 vaccines are substantially impaired in patients receiving inhibitors of Bruton tyrosine kinase, Janus kinase, or Bcl-2, or antibodies targeting CD20 or CD38.^5–10^ This data are in line with our results, where only one patient on anti-CD20 therapy mounted a weak antibody response (titer of 7.2 BAU/ml), despite complete absence of measurable circulating CD19^+^ B cells (CD19^+^Bc). The remaining n = 8 individuals being currently treated (CD20-antibodies or Bcl-2-inhibitor) did not mount a measurable humoral response.

**Figure 2.**
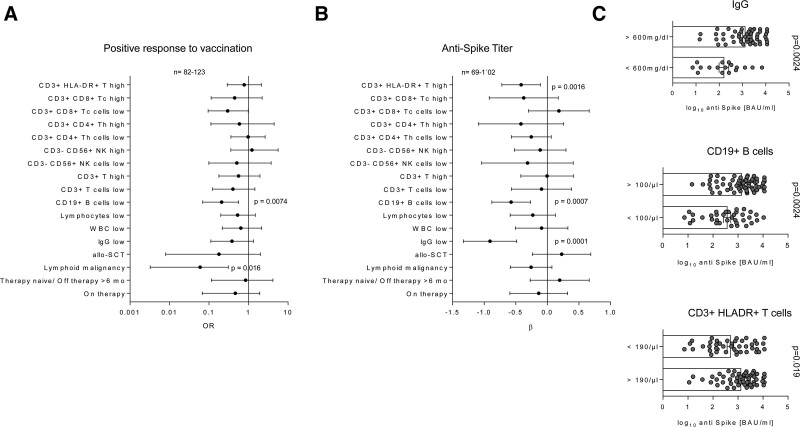
**Univariable modeling of the positive vaccination response and post-vaccination antibody titer.** Correlation of the candidate factors affecting the probability of positive vaccination response (A, > 7 BAU/mL) and antispike antibody titer in the individuals with positive antibody response (B) was investigated by a series of univariable logistic and linear regression models, respectively. Significance of the model estimates was determined by Wald Z test (logistic regression: OR/odds ratio) or T test (linear regression: β), as appropriate, and corrected for multiple testing with Benjamini-Hochberg method. Estimate values with 95% confidence intervals are presented in Forest plots. Ranges of complete observations are indicated under the plots. (C) Antispike antibody titer in the individuals with positive antibody response was investigated in the study participants stratified by pre-vaccination circulating IgG, CD19^+^B cells and CD3^+^HLA-DR^+^ T-cells levels. Statistical significance was determined by two-tailed T test. Test p values are presented in the plot captions. allo-SCT = allogeneic stem cell transplantation; B = B cells; IgG = immunoglobulin G; LYM = lymphoid malignancy; NK = natural killer cells; T = T cells; Tc = cytotoxic T-cells; Th = T helper cells; WBC = white blood cells.

We next sought to define easy to measure immunological predictors for vaccine-responders. To this end, qualitative and quantitative analysis of the lymphocyte subpopulations in the peripheral blood in form of a cellular immune profile was carried out using a Canto II flow cytometer and lyophilized 8-color tubes from Becton Dickinson (for details, see Suppl. Digital File). Univariable logistic modeling revealed that the probability of vaccination response is negatively influenced by low CD19^+^Bc (<100/µL; OR: 0.301 95% CI 0.07–0.562; Figure 2A, Suppl. Table S3). This result was substantiated by univariate linear modeling of the antispike levels in individuals with positive antibody response: low CD19^+^Bc (β: −0.57, 95% CI −0.88 to −0.27) and also low IgG values (<600 mg/dL, β: −0.91, 95% CI −1.3 to −0.48) and high HLA-DR3^+^ T-cells (>190/µL, β: −0.42, 95% CI −0.72 to 0.11) are negatively linked to antibody titer height (Figure 2B and C). Importantly, lymphoid malignancy (OR = 0.077), bone marrow transplantation (OR = 0.16), and low CD19^+^ Bc counts were identified independent negative predictors of vaccination response in the study cohort identified by LASSO (least absolute shrinkage and selection operator) multivariate modeling (for details, see Suppl. Digital Methods and Table S4). Furthermore, elevated counts of T cells and low circulating NK cell levels were associated with the lower probability of the vaccination response in the multivariate setting (Suppl. Table S4). In turn, low circulating CD19^+^Bc and low IgG were the sole independent unfavorable correlates of antispike titers in the multivariate analysis (Suppl. Table S4). These results are partly in contrast to results obtained by Benda et al,^11^ reporting a negative influence of low lymphocyte but also an influence of lower NK-cell counts on vaccination response. Importantly, the multiparameter LASSO model displayed a superior sensitivity (0.8) and accuracy (area under the curve = 0.92 [0.86–0.98]) at predicting the vaccination response over single risk factors such as Bc levels or malignancy type (Suppl. Figure S3A). The multiparameter LASSO model was also substantially better at predicting the antibody titer than IgG and CD19^+^ Bc levels alone (Suppl. Figure S3). Collectively, this suggest that comprehensive immunoprofiling may help to identify vaccination responders amongst hematological patients.

Patients receiving B-cell-targeting agents and having reduced or even absent circulating CD19^+^Bc are particularly prone to insufficient antibody responses. Thus, we took a more detailed look into patients with low CD19^+^Bc. Of these 50 patients, 35 patients were responder (titer > 7 BAU/mL), which is in line with rheumatological patients receiving rituximab, showing that once peripheral CD19^+^Bc are present (even at very low amounts of 1%), a humoral vaccination response can be mounted.^12^ However, data interpretations are limited by the lack of knowledge how peripheral CD19^+^Bc reflect CD19^+^Bc and plasma cell abundance in the bone marrow. Notably, individual patients (COAK_45) mounted an antibody titer (37.6 BAU/mL) even though circulating CD19^+^Bc were absent. This patient, after rituximab treatment until June 2017 and idelalisib until October 2019 currently undergoes PD-1 antibody therapy for metastatic skin cancer. Checkpoint inhibitor therapies may impact serological outcome after vaccination,^13^ whereas in our patient, it may explain the humoral response even in the absence of CD19^+^Bc.

In summary, our data put forward lymphoid malignancy, low CD19^+^Bc and IgG values as clinically applicable predictors for an insufficient immune response to SARS-CoV-2 vaccination and characteristic of the patient subset requiring dense serological monitoring, boosting vaccination and additional preventive measures. Moreover, hematological patients without humoral immune response to the SARS-CoV-2 vaccine may mount T cell-mediated immunity.^14^ Future studies will demonstrate how the subsequent booster-vaccination(s) may overcome insufficient humoral responses particularly in patients with lymphoid diseases and how efficient (fully) vaccinated hematological patients are protected from severe COVID-19 as the humoral response is only one of the manifold adaptive and innate immunological mechanisms induced by vaccines.^15^ Finally, the great majority of the investigated individuals mounted a measurable humoral response to the immunization despite ongoing therapy or lymphoid disease. Hence, we advise all patients to be vaccinated irrespective of their underlying malignancy type, their therapy and immune status.

## ACKNOWLEDGMENTS

The authors declare to external funding sources. Figure S1 was created with BioRender.com.

## AUTHOR CONTRIBUTIONS

VP, NS, SS, and DW contributed equally.

## DISCLOSURES

PT owns a data science enterprise DAAS Tirol. All the other authors have no conflicts of interest to disclose.

## Supplementary Material



## References

[1] Mehta V Goel S Kabarriti R. Case fatality rate of cancer patients with COVID-19 in a New York Hospital system. Cancer Discov. 2020;10:935–941. 32357994 10.1158/2159-8290.CD-20-0516 PMC7334098

[2] Vijenthira A Gong IY Fox TA. Outcomes of patients with hematologic malignancies and COVID-19: a systematic review and meta-analysis of 3377 patients. Blood. 2020;136:2881–2892. 33113551 10.1182/blood.2020008824 PMC7746126

[3] Toyoshima Y Nemoto K Matsumoto S. SARS-CoV-2 genomic variations associated with mortality rate of COVID-19. J Hum Genet. 2020;65:1075–1082. 32699345 10.1038/s10038-020-0808-9 PMC7375454

[4] Nagy Á Pongor S Győrffy B. Different mutations in SARS-CoV-2 associate with severe and mild outcome. Int J Antimicrob Agents. 2021;57:106272. 33347989 10.1016/j.ijantimicag.2020.106272 PMC7755579

[5] Crombie JL Sherman AC Cheng C-A. Activity of mRNA COVID-19 vaccines in patients with lymphoid malignancies. Blood Adv. 2021;5:3062–3065. 34387646 10.1182/bloodadvances.2021005328 PMC8362656

[6] Herishanu Y Avivi I Aharon A. Efficacy of the BNT162b2 mRNA COVID-19 vaccine in patients with chronic lymphocytic leukemia. Blood. 2021;137:3165–3173. 33861303 10.1182/blood.2021011568 PMC8061088

[7] Perry C Luttwak E Balaban R. Efficacy of the BNT162b2 mRNA COVID-19 vaccine in patients with B-cell non-Hodgkin lymphoma. Blood Adv. 2021;5:3053–3061. 34387648 10.1182/bloodadvances.2021005094 PMC8362658

[8] Maneikis K Šablauskas K Ringelevičiūtė U. Immunogenicity of the BNT162b2 COVID-19 mRNA vaccine and early clinical outcomes in patients with haematological malignancies in Lithuania: a national prospective cohort study. Lancet Haematol. 2021;8:e583–e592. 34224668 10.1016/S2352-3026(21)00169-1 PMC8253543

[9] Harrington P Doores KJ Radia D. Single dose of BNT162b2 mRNA vaccine against severe acute respiratory syndrome coronavirus-2 (SARS-CoV-2) induces neutralising antibody and polyfunctional T-cell responses in patients with chronic myeloid leukaemia. Br J Haematol. 2021;194:999–1006. 34085278 10.1111/bjh.17568 PMC8239833

[10] Pimpinelli F Marchesi F Piaggio G. Fifth-week immunogenicity and safety of anti-SARS-CoV-2 BNT162b2 vaccine in patients with multiple myeloma and myeloproliferative malignancies on active treatment: preliminary data from a single institution. J Hematol Oncol. 2021;14:81. 34001183 10.1186/s13045-021-01090-6 PMC8128283

[11] Benda M Mutschlechner B Ulmer H. Serological SARS-CoV-2 antibody response, potential predictive markers and safety of BNT162b2 mRNA COVID-19 vaccine in haematological and oncological patients. Br J Haematol. 2021;195:523–531. 34346068 10.1111/bjh.17743 PMC8444745

[12] Mrak D Tobudic S Koblischke M. SARS-CoV-2 vaccination in rituximab-treated patients: B cells promote humoral immune responses in the presence of T-cell-mediated immunity. Ann Rheum Dis. 2021;80:1345–1350. 34285048 10.1136/annrheumdis-2021-220781

[13] Ligumsky H Safadi E Etan T. Immunogenicity and safety of the BNT162b2 mRNA COVID-19 vaccine among actively treated cancer patients [Epub ahead of print. August, 2021]. JNCI J Natl Cancer Inst. doi: 10.1093/jnci/djab174 10.1093/jnci/djab174 PMC8499747 34453830

[14] Marasco V Carniti C Guidetti A. T-cell immune response after mRNA SARS-CoV-2 vaccines is frequently detected also in the absence of seroconversion in patients with lymphoid malignancies. Br J Haematol. 2022;196:548 558. 34649298 10.1111/bjh.17877 PMC8653177

[15] Mairhofer M Kausche L Kaltenbrunner S. Humoral and cellular immune responses in SARS-CoV-2 mRNA-vaccinated patients with cancer. Cancer Cell. 2021;39:1171–1172. 34450047 10.1016/j.ccell.2021.08.001 PMC8367743

