# Correction Notice: Serologic Responses to COVID-19 Vaccines in Hematological Patients Are Predominantly Impaired in Lymphoid but not in Myeloid Malignancies

**DOI:** 10.1097/HS9.0000000000000731

**Published:** 2022-05-12

Since the publication of the article entitled “Serologic Responses to COVID-19 Vaccines in Hematological Patients Are Predominantly Impaired in Lymphoid but not in Myeloid Malignancies” (*HemaSphere.* 2022;6(3):e686), the authors have requested a spelling correction for author Christian Irsara. In the original publication, his surname, Irsara, was missing one letter. This has now been adjusted.

The changes have been made online: https://journals.lww.com/hemasphere/Fulltext/2022/03000/Serologic_Responses_to_COVID_19_Vaccines_in.3.aspx

