# Supplementary material for: Serologic Responses to COVID-19 Vaccines in Hematological Patients Are Predominantly Impaired in Lymphoid but not in Myeloid Malignancies
Source: Hemasphere. 2022 Feb 15;6(3):e686. doi: 10.1097/HS9.0000000000000686 (PMC8849383; doi:10.1097/HS9.0000000000000686)
Supplement: Supplementary file 1 [file hs9-6-e686-s001.docx]

**Supplemental File**

**Serologic responses to COVID-19 vaccines in hematological patients are pre-dominantly impaired in lymphoid but not in myeloid malignancies**

Verena Petzer^1,†^, Normann Steiner^1,†^, Olga Angelova-Unterberger^1^, Gabriele Hetzenauer^1^, [Kathrin Philipp-Abbrederis](https://pubmed.ncbi.nlm.nih.gov/?sort=date&term=Abbrederis+K&cauthor_id=24979177)^1^, Ella Willenbacher^1^, Clemens Feistritzer^1^, Wolfgang Willenbacher^1^, Jakob Rudzki^1^, Reinhard Stauder^1^, Florian Kocher^1^, Andreas Seeber^1^, Andreas Pircher^1^, Piotr Tymoszuk^2^, Christian Isara^3^, Alexander Egger^3^, Vilmos Fux^3^, Markus Anliker^3^, Eberhard Gunsilius^1^, David Nachbaur^1^, Stefan Schmidt^1,†^ and Dominik Wolf ^1,†^

^1^ Department of Internal Medicine V (Hematology and Medical Oncology), Medical University of Innsbruck, Anichstraße 35, A-6020 Innsbruck, Austria;

^2^ Data Analytics As a Service Tirol, DAAS Tirol, Innsbruck, Austria

^3^ Central Institute of Clinical Chemistry and Laboratory Medicine Medical University of Innsbruck, Austria.

*† Contributed equally*

Supplemental Methods

Study design

Baseline laboratory assessment and analysis of immune status *via* flow cytometry, measurement of total IgG and anti-SARS-CoV-2 antibodies against viral spike protein (RBD, Abbott, WHO) was analyzed up to 50 days prior to vaccination (median: 2 days, IQR: ) prior to the first dose and follow up was performed up to 85 days after the second dose (median 35 days, IQR: 25-52 days). Details on the experimental setup are shown in Figure S1B. The trial protocol was approved by the institutional review board at the Medical University of Innsbruck (approval number: 1331/2021).

Laboratory analyses and complete blood count were assessed by standard methods as part of patient care at the Central Institute of Clinical Chemistry and Laboratory Medicine Medical University of Innsbruck.

Anti-SARS-CoV-2 antibody assay

Antibodies against the nucleocapsid (N) protein and the receptor-binding domain (RBD) of the spike (S) protein of SARS-CoV-2 were detected in serum using the Roche Elecsys Anti-SARS-CoV-2 assay on the Cobas e602 platform (Roche Diagnostics, Rotkreuz, Switzerland) and the Abbott SARS-CoV-2 IgG II Quant assay on the ARCHITECT i platform (Abbott Laboratories Abbott Park, IL, USA), respectively. The results of the N protein-based assay are reported qualitatively (positive, negative), whereas the results of the S protein-based assay are reported quantitatively, given in binding antibody units per millilitre (BAU/ml) in reference to the first WHO International standard for human anti-SARS-CoV-2-immunoglobulin (NIBSC code: 20/136) (1). All samples were processed according to the manufacturers’ procedures with the specified controls and calibrators by trained laboratory staff.

Cellular immune profile using flow cytometry:

In the present study the qualitative and quantitative analysis of the lymphocyte subpopulations in the peripheral blood in form of a cellular immune profile (synonym: lymphocyte subpopulation typing) was carried out using a Canto II flow cytometer and lyophilized 8-color tubes from Becton Dickinson (BD). Peripheral blood was analyzed according to Euroflow’s standards and instructions (2). The following antibodies were used: CD45 (V500C), CD3 (APC), CD4 (PerCPCy5.5), CD8 (FITC), HLA-DR (V450) and CD19 (PECy7), CD14 (APC H7), CD56 (PE). In total 30,000 events were acquired. Leucocytes, lymphocytes, B cells (CD19+), T cells (CD3+), NK cells (CD3-, CD56+), T helper cells (CD3+, CD4+), T suppressor cells/cytotoxic cells (CD3+CD8+), CD4/CD8 ratio, double positive T cells (CD3+, CD4+, CD8+), double negative T cells (CD3+, CD4-CD8-), NKT (cytotoxic) cells (CD3+, CD56+), and activated T cells (CD3+, HLADR+) were allocated.

Statistical analysis

*Data transformation and visualization*

Data transformation, statistical analysis and analysis result visualization was done with R version 4.0.5, tidyverse environment and cowplot package (3–5). Immunoglobulin G concentration and the counts of circulating B, T and NK cell populations prior to vaccination were stratified by standard cutoffs. The normal ranges of CD3+ CD16+ CD56+ NKT cell counts were obtained from (6) . The normal ranges of CD3+ HLA-DR+ T cell counts were obtained from (7). Positive vaccination response was defined with a > 7 BAU/ml cutoff. The anti-spike blood antibody concentration in the participants with a positive vaccination response was log_10_ transformed to improve normality as determined by Shapiro-Wilk test (Supplemental Figure S4). The list of study variables and their stratification schemes are listed in Table S2.

*Hypothesis testing and multiple comparison adjustment*

Statistical significance of differences in frequency of positive vaccination response between analysis groups was determined with χ2 test. Statistical significance of differences in log_10_-transformed anti-S1/S2 antibody concentrations between analysis groups was assessed by two-tailed T test or one-way ANOVA depending on the group number. Test and modeling p values were corrected for multiple comparisons with Benjamini- Hochberg method.6 The home-developed tools for hypothesis testing based on the base R functions are available from https://github.com/PiotrTymoszuk/counting-tools.

*Univariable modeling*

Correlation of the malignancy type, anti-cancer therapy status, pre-vaccination immunoglobulin G concentrations and pre-vaccination levels of circulating B, T and NK cell subsets with the chance of positive vaccination response and log10-transformed antibody titer in the participants with positive vaccination response was accomplished with a series of univariable logistic (vaccination response) or linear (titer) regression models. Significance of the model estimates was determined by Wald Z test (logistic regression: OR/odds ratio) or T test (linear regression: β), as appropriate, and corrected for multiple testing with Benjamini-Hochberg method (8). Model quality control was done by visual inspection of the residuals vs. fitted and residuals QQ plots. The modeling and quality check tasks were accomplished with home-developed wrappers around base R lm() and glm() function and tools provided by broom package (9) (available from https://github.com/PiotrTymoszuk/lm_qc_tools). The complete results of univariate modeling are presented in Supplemental Table S4.

*Multivariable modeling*

Multivariate modeling of the positive vaccination response and log10-transformed antibody titer in the participants with positive vaccination response as a function of the malignancy type, anti-cancer therapy status, pre-vaccination immunoglobulin G concentrations and pre-vaccination levels of circulating B, T and NK cell subsets was done with LASSO (least absolute shrinkage and selection operator) logistic (vaccination response) or linear regression (titer) (10). LASSO models were constructed using cv.glmnet() function from glmnet package (11) and validated by least-one-out cross-validation (LOOCV) using caret package (12). Visual mode quality control was done as described above for univariable modeling. Performance of the multi-parameter LASSO model and univariate logistic models at predicting vaccination response was tested with receiver-operating characteristic (ROC) using tools provided by optimaCutpoint package (13). Correlation of the antibody titer predicted by the multi-parameter LASSO model and univariate linear models with the actual antibody titers was investigated by Spearman test. The results of multivariate modeling (non-zero estimates, redistribution and cross-validation errors) and performance testing are presented in Supplementary Table S4 and Figure S3.

Supplemental Figures


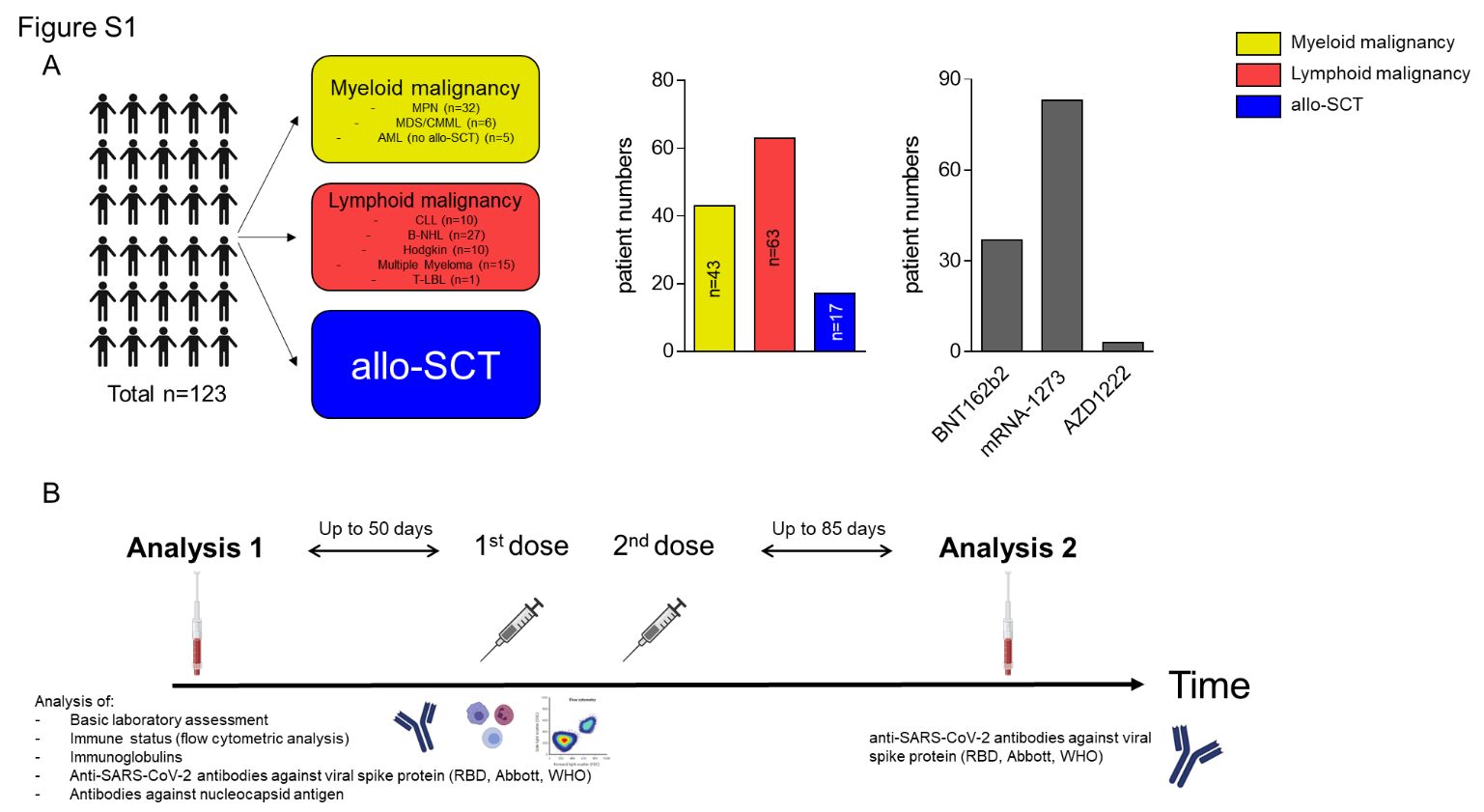


**Figure S1.** (A) Overview of disease groups, patient numbers and vaccination applied. (B) Timeline and schematic overview of analysis.


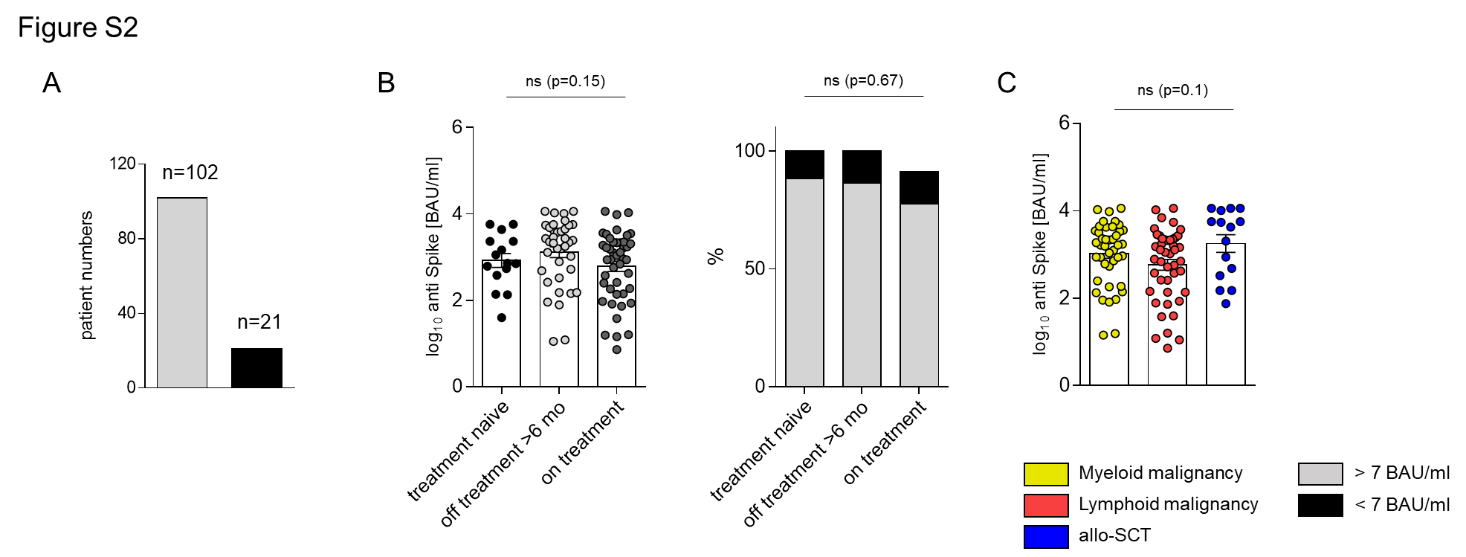


**Figure S2.** (A) Frequency of patients with a serological response according to BAU/ml at timepoint ‘Analysis 2’ (see Figure S1B). (B, C) Frequency of positive vaccination response and anti-spike antibody titer in the individuals with positive antibody response was investigated in the study participants stratified by therapy status and type of malignancy, respectively. Statistical significance was determined by or one-way ANOVA and corrected for multiple testing with Benjamini-Hochberg method. Test p values are presented in the plot. BAU: binding antibody units.


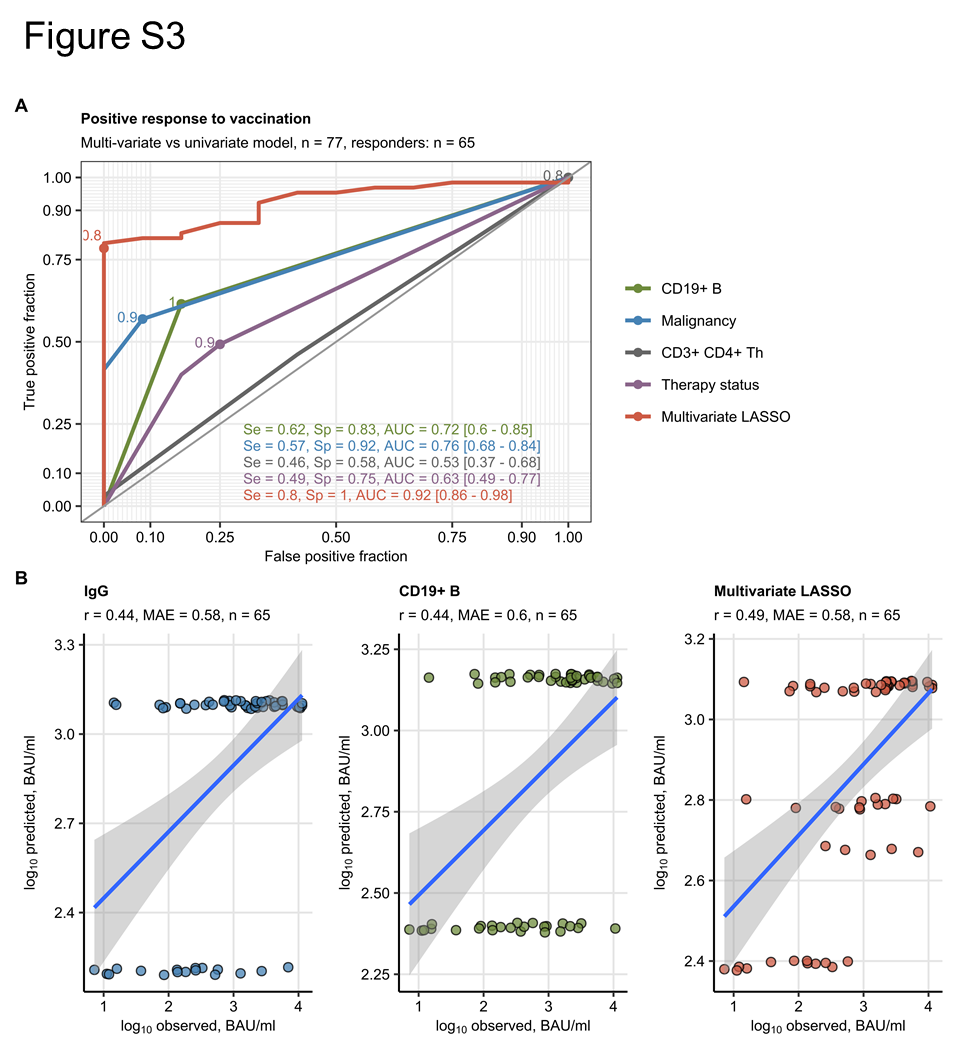


**Figure S3. Prediction of positive vaccination response by multi-parameter LASSO modeling.**

Multi-parameter LASSO (least absolute shrinkage and selection operator) models predicting positive vaccination response (> 7 BAU/ml, logistic regression) and log10 anti-S1/S2 antibody titer (linear regression) were constructed and validated by leave-one-out cross-validation (LOOCV) in the study cohort subset with the complete data record. Performance of the LASSO model and univariate models including the strongest co-variates of positive vaccination response and antibody titers was compared by receiver-operating characteristic (ROC) and Spearman correlation. Numbers of complete observations and vaccination responders are presented in the plots.

**(A)** Model performance at predicting the positive vaccination response tested by ROC. Line and text color codes for model type. Sensitivity (Se), specificity (Sp) ans area under the curve (AUC) with 95% confidence intervals are shown in the plot.

**(B)** Model performance at predicting antibody levels tested by Spearman correlation. Each point represents a single observation, fittted linear trends with 95% confidence regions are shown as blue lines with gray ribbons. Correlation coefficient (t), mean-absolute error (MAE) are shown in the plot captions.


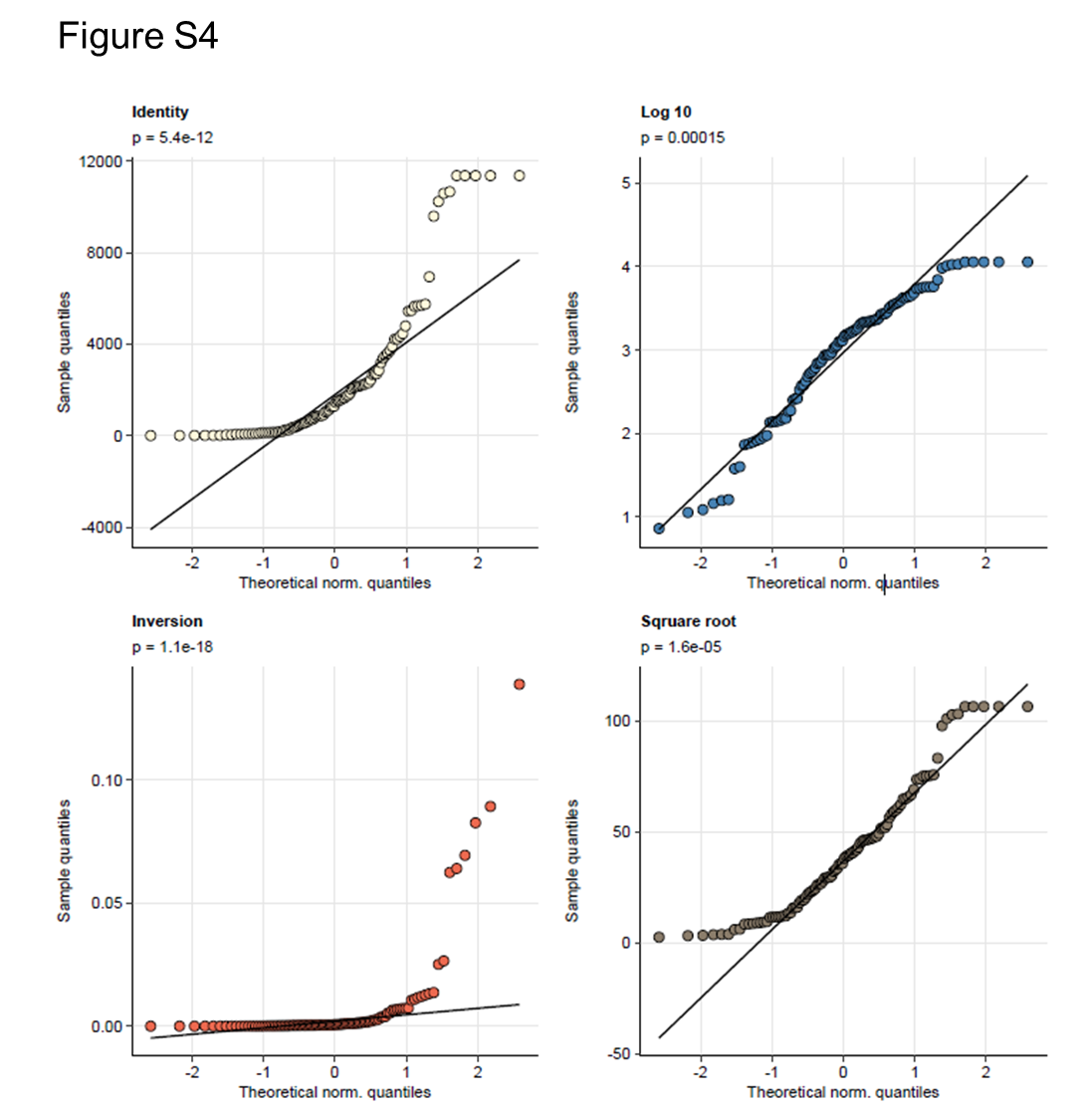


**Figure S4.** Normality of the anti-S1/S2 antibody titer after transformation. Anti-S1/S2 antibody titer in the individuals with positive antibody response (> 7 BAU/ml) was transformed with log_10_, inversion and square root functions and the normality was assessed by Shapiro-Wilk test. Variable quantiles and theoretical normal distribution quantliles for each transformation were presented in QQ plots. Normality test p value in displayed in the plot captions.

Supplemental Tables

**Table S1.** Characteristics of Study Cohort.

| **Characteristics** | **All** | **Myeloid** | **Lymphoid** | **Allo-SCT** |
| --- | --- | --- | --- | --- |
| Number of Patients, n (%) | 123 | 43 (35)  MPN: 32 (26)  AML: 5 (4.1)  MDS/CMML: 6 (4.9) | 63 (51,2)  Multiple Myeloma: 15 (12.2)  Hodgkin: 10 (8.1)  B-NHL: 27 (22)  CLL: 10 (8.1)  T-LBL: 1 (0.8) | 17 (13,8) |
| Male/Female, n | 55/68 | 16/27 | 34/29 | 5/12 |
| Median Age (years) | 65 [20-90] | 67 [31-86] | 66 [20-90] | 52 [30-74] |
| Vaccination BNT162b2/ mRNA-1273 / AZD1222 | 37/83/3 | 8/33/2 | 18/45/0 | 11/5/1 |

**Table S2.** The list of study variables and their stratification schemes.

| **Variable label** | **Unit** | **Stratification cutoff** |
| --- | --- | --- |
| IgG | mg/dl | 600 |
| WBC | cells/µl | 4000 |
| Lymphocytes | cells/µl | 1000 |
| CD19+ B | cells/µl | 100 |
| CD3+ T | cells/µl | 500;1600 |
| CD3- CD56+ NK | cells/µl | 50;400 |
| CD3+ CD4+ Th | cells/µl | 500;1200 |
| CD3+ CD8+ Tc | cells/µl | 150;1000 |
| CD3+ CD16+ CD56+ NKT | cells/µl | 20;350 |
| CD3+ HLA-DR+ T | cells/µl | 190 |

**Table S3. Results of univariate modeling.**

| **Response** | **Model type** | **Independent variable** | **Level** | **N** | **Estimate** | **Significance** |
| --- | --- | --- | --- | --- | --- | --- |
| AB response | logistic regression | Therapy status | on | 119 | OR = 0.462 [0.0672 - 1.93] | ns (p = 0.46) |
| AB response | logistic regression | Therapy status | off | 119 | OR = 0.844 [0.115 - 4.16] | ns (p = 0.88) |
| AB response | logistic regression | Malignancy | LYM | 123 | OR = 0.0595 [0.00324 - 0.308] | p = 0.016 |
| AB response | logistic regression | Malignancy | BMT | 123 | OR = 0.179 [0.00795 - 1.99] | ns (p = 0.28) |
| AB response | logistic regression | IgG | low | 82 | OR = 0.381 [0.111 - 1.33] | ns (p = 0.23) |
| AB response | logistic regression | WBC | low | 123 | OR = 0.64 [0.216 - 2.16] | ns (p = 0.54) |
| AB response | logistic regression | Lymphocytes | low | 123 | OR = 0.528 [0.198 - 1.47] | ns (p = 0.32) |
| AB response | logistic regression | CD19+ B | low | 123 | OR = 0.209 [0.0693 - 0.562] | p = 0.0074 |
| AB response | logistic regression | CD3+ T | low | 123 | OR = 0.403 [0.123 - 1.45] | ns (p = 0.25) |
| AB response | logistic regression | CD3+ T | high | 123 | OR = 0.558 [0.178 - 1.95] | ns (p = 0.46) |
| AB response | logistic regression | CD3- CD56+ NK | low | 123 | OR = 0.506 [0.0991 - 3.75] | ns (p = 0.54) |
| AB response | logistic regression | CD3- CD56+ NK | high | 123 | OR = 1.22 [0.356 - 5.61] | ns (p = 0.83) |
| AB response | logistic regression | CD3+ CD4+ Th | low | 123 | OR = 0.978 [0.359 - 2.64] | ns (p = 0.97) |
| AB response | logistic regression | CD3+ CD4+ Th | high | 123 | OR = 0.587 [0.112 - 4.44] | ns (p = 0.64) |
| AB response | logistic regression | CD3+ CD8+ Tc | low | 123 | OR = 0.296 [0.0946 - 0.981] | ns (p = 0.077) |
| AB response | logistic regression | CD3+ CD8+ Tc | high | 123 | OR = 0.444 [0.113 - 2.21] | ns (p = 0.4) |
| AB response | logistic regression | CD3+ HLA-DR+ T | high | 121 | OR = 0.778 [0.289 - 2.12] | ns (p = 0.69) |
| AB titer | linear regression | Therapy status | on | 98 | beta = -0.136 [-0.593 - 0.32] | ns (p = 0.65) |
| AB titer | linear regression | Therapy status | off | 98 | beta = 0.195 [-0.272 - 0.662] | ns (p = 0.52) |
| AB titer | linear regression | Malignancy | LYM | 102 | beta = -0.257 [-0.587 - 0.0722] | ns (p = 0.22) |
| AB titer | linear regression | Malignancy | BMT | 102 | beta = 0.226 [-0.236 - 0.687] | ns (p = 0.47) |
| AB titer | linear regression | IgG | low | 69 | beta = -0.908 [-1.33 - -0.485] | p = 0.00014 |
| AB titer | linear regression | WBC | low | 102 | beta = -0.0913 [-0.507 - 0.325] | ns (p = 0.72) |
| AB titer | linear regression | Lymphocytes | low | 102 | beta = -0.231 [-0.589 - 0.127] | ns (p = 0.32) |
| AB titer | linear regression | CD19+ B | low | 102 | beta = -0.573 [-0.879 - -0.266] | p = 0.00074 |
| AB titer | linear regression | CD3+ T | low | 102 | beta = -0.0927 [-0.567 - 0.382] | ns (p = 0.73) |
| AB titer | linear regression | CD3+ T | high | 102 | beta = -0.00454 [-0.42 - 0.411] | ns (p = 0.98) |
| AB titer | linear regression | CD3- CD56+ NK | low | 102 | beta = -0.313 [-1.04 - 0.409] | ns (p = 0.52) |
| AB titer | linear regression | CD3- CD56+ NK | high | 102 | beta = -0.114 [-0.523 - 0.295] | ns (p = 0.65) |
| AB titer | linear regression | CD3+ CD4+ Th | low | 102 | beta = -0.255 [-0.571 - 0.0617] | ns (p = 0.21) |
| AB titer | linear regression | CD3+ CD4+ Th | high | 102 | beta = -0.418 [-1.09 - 0.254] | ns (p = 0.32) |
| AB titer | linear regression | CD3+ CD8+ Tc | low | 102 | beta = 0.182 [-0.299 - 0.662] | ns (p = 0.55) |
| AB titer | linear regression | CD3+ CD8+ Tc | high | 102 | beta = -0.374 [-0.92 - 0.172] | ns (p = 0.29) |
| AB titer | linear regression | CD3+ HLA-DR+ T | high | 102 | beta = -0.415 [-0.72 - -0.111] | p = 0.016 |

**Table S4. Results of multivariable modeling.**

| **Response** | **Method** | **N total** | **N responders** | **Lambda** | **MAE** | **MAE(CV)** | **Variable** | **Estimate** |
| --- | --- | --- | --- | --- | --- | --- | --- | --- |
| Vaccination response | LASSO logistic regression | 77 | 65 | 0,014 | 0,19 | 0,24 | Intercept | OR = 59 |
|  |  |  |  |  |  |  | Therapy status:off | OR = 2.6 |
|  |  |  |  |  |  |  | Malignancy:LYM | OR = 0.077 |
|  |  |  |  |  |  |  | Malignancy:BMT | OR = 0.16 |
|  |  |  |  |  |  |  | WBC:low | OR = 0.76 |
|  |  |  |  |  |  |  | CD19+ B:low | OR = 0.2 |
|  |  |  |  |  |  |  | CD3+ T:high | OR = 0.51 |
|  |  |  |  |  |  |  | CD3- CD56+ NK:low | OR = 1.2 |
|  |  |  |  |  |  |  | CD3+ CD4+ Th:low | OR = 2.7 |
|  |  |  |  |  |  |  | CD3+ CD8+ Tc:low | OR = 0.37 |
|  |  |  |  |  |  |  | CD3+ CD8+ Tc:high | OR = 0.78 |
|  |  |  |  |  |  |  | CD3+ HLA-DR+ T:high | OR = 1.1 |
| log_10_ anti-S1/S2 | LASSO linear regression | 65 | NA | 0,16 | 0,58 | 0,61 | Intercept | β = 3.1 |
|  |  |  |  |  |  |  | IgG:low | β = -0.4 |
|  |  |  |  |  |  |  | CD19+ B:low | β = -0.29 |

Lambda: model shrinkage parameter lambda, MAE: mean absolute error in the training data, MAE(CV): mean absolute error in leave-one-out cross-validation. LYM: lymphoid, BMT: bone marrow transplantation, CD19^+^ B: CD19^+^ B cells, IgG: immunoglobulin G, WBC: white blood cell count, OR: odds ratio

**References**

1. WHO/BS.2020.2403 Establishment of the WHO International Standard and Reference Panel for anti-SARS-CoV-2 antibody [Internet].

2. Van Dongen JJM, Lhermitte L, Böttcher S, Almeida J, Van Der Velden VHJ, Flores-Montero J, et al. EuroFlow antibody panels for standardized n-dimensional flow cytometric immunophenotyping of normal, reactive and malignant leukocytes. 2012 May 3;26(9):1908–75.

3. Bebeau C. Graphicacy for Numeracy: Review of Fundamentals of Data Visualization: A Primer on Making Informative and Compelling Figures by Claus O. Wilke (2019). 2019;11(1).

4. Wickham H. Getting started with qplot BT-ggplot2: elegant graphics for data analysis. 2009;9–26.

5. Wickham H, Averick M, Bryan J, Chang W, D’ L, Mcgowan A, et al. Welcome to the Tidyverse. 2019 Nov 21;4(43):1686.

6. Melzer S, Zachariae S, Bocsi J, Engel C, Löffler M, Tárnok A. Reference intervals for leukocyte subsets in adults: Results from a population-based study using 10-color flow cytometry. 2015 Jul 1;88(4):270–81.

7. Bisset LR, Lung TL, Kaelin M, Ludwig E, Dubs RW. Reference values for peripheral blood lymphocyte phenotypes applicable to the healthy adult population in Switzerland. 2004 Mar;72(3):203–12.

8. Benjamini Y, Hochberg Y. Controlling the False Discovery Rate: A Practical and Powerful Approach to Multiple Testing. 1995 Jan 1;57(1):289–300.

9. Robinson D. broom: An R Package for Converting Statistical Analysis Objects Into Tidy Data Frames. 2014 Dec 11;

10. Tibshirani R. Regression Shrinkage and Selection Via the Lasso. 1996 Jan 1;58(1):267–88.

11. Friedman J, Hastie T, Tibshirani R. Regularization paths for generalized linear models via coordinate descent. 2010 Feb 2;33(1):1–22.

12. Kuhn M. Building predictive models in R using the caret package. Journal of Statistical Software. 2008;28(5):1-26.

13. López-Ratón M, Rodríguez-Álvarez MX, Cadarso-Suárez C, Gude-Sampedro F. Optimalcutpoints: An R package for selecting optimal cutpoints in diagnostic tests. Journal of Statistical Software. 2014;61(8):1-36.
